# Analysis of pathogenic variants in retinoblastoma reveals a potential gain of function mutation

**DOI:** 10.18632/genesandcancer.239

**Published:** 2025-01-20

**Authors:** Ana María Peña-Balderas, Mayra Martínez-Sánchez, Isaí Olmos-Sánchez, Karla Calderón-González, Mariana Moctezuma-Dávila, Martha Rangel-Charqueño, Jesús Hernández-Monge, Vanesa Olivares-Illana

**Affiliations:** ^1^Laboratorio de Interacciones Biomoleculares y Cáncer, Instituto de Física Universidad Autónoma de San Luis Potosí, San Luis Potosí 78210, México; ^2^División de Cirugía, Departamento de Oftalmología, Hospital Central “Ignacio Morones Prieto”, San Luis Potosí, México; ^3^Investigador por México, Laboratorio de Biomarcadores Moleculares, Instituto de Física, Universidad Autónoma de San Luis Potosí, México City, México; ^4^Present address: Houston Methodist Hospital, Department of Pathology and Genomic Medicine, Houston, TX 77030, USA

**Keywords:** retinoblastoma, cancer, gain of function, mutants, pathogenic variants

## Abstract

*Retinoblastoma (Rb1)* is a gene that codes for a tumour suppressor protein involved in various types of cancer. It was first described in retinoblastoma and is segregated as an autosomal dominant trait with high penetrance. In 1971, Knudson proposed his hypothesis of the two hits, where two mutational events are required to initiate tumour progression. We analysed three different point mutations present in patients’ retinoblastoma. We produced three cell lines with retinoblastoma protein (RB) mutated in various regions: the missense pN328H, pD718N, and the nonsense early stop codon pR552*. We studied the effect of these point mutations on levels of mRNA and protein expression, proliferation, viability, localisation, and migration using an RBKO cell line. All three affected their localisation patterns and proliferation. However, the pR552* mutation also increases viability and migration. Moreover, when this mutation is simultaneously expressed with a wild-type RB, the phenotype and proliferation parameters are as with the mutant alone, suggesting that maybe only one mutated allele is needed to trigger the characteristic cancer phenotype. In other words, the pR552* mutant behaves more like a gain-of-function or oncogenic mutant. Indeed, a family carrying this mutation showed complete penetrance and high expressivity.

## INTRODUCTION

Retinoblastoma, also known as retina cancer, is a rare pediatric cancer that occurs among children aged 0 to 5 years old. It is the most frequent ocular cancer, accounting for 4% of all pediatric neoplasms [1]. Nevertheless, the incidence varies depending on the region; for example, in non-developed countries, it can increase up to 24 cases per million of habitants [2, 3]. Retinal tumours’ are commonly detected by visually noticeable signs such as leukocoria (white pupil) or strabismus (misaligned eyes), different-coloured irises, and poor vision, among others [4]. Retinoblastoma is curable as long as it is detected and treated early; if not, the tumour tends to invade and destroy internal structures of the eye globe, eventually leading to the development of metastasis and death [5]. The *Rb1* gene was the first tumour suppressor identified and cloned; its association with the development of retinal cancer gave rise to its name, “RB”, for retinoblastoma [6]. Although the loss of *Rb1* plays a central role in the development of retinoblastoma, other genes are also protagonists, such as Myc-N, Mdm2, Mdmx, p53, and others recently identified [5, 7].

Many authors have annotated several alterations in the *Rb1* gene, not only for retinoblastoma but also for other cancers [8–11]. The mutations reported on the *Rb1* gene are widely distributed across all 27 exons and their regulatory regions [12]. Missense variants, synonymous mutants, early stop codons (nonsense), and deletions, among others, have been reported [13]. More than one hundred different mutations have been identified and reported in the *Rb1* gene associated with retinoblastoma in the COSMIC database: https://cancer.sanger.ac.uk/cosmic [14]. It has been suggested that unlike other types of cancers, retinoblastoma tumours do not exhibit large chromosomal rearrangements but rather punctual changes scattered throughout the gene [4]. Not all mutations cause the same phenotype; for instance, it has been observed that early stop codons are associated with high penetrance of the disease; missense mutations, small in-frame indels, promoter and some splicing mutations are associated with low penetrance phenotype [11]. The RB protein, encoded by the *Rb1* gene, is a large polypeptide consisting of 928 amino acid residues that undergo post-translational modifications. It comprises four domains with intrinsically disordered regions, providing the protein with significant plasticity. These characteristics allow RB to have a broad range of interaction partners [15, 16]. This network of interactions, where RB functions as a hub, allows RB to be involved in many different pathways. A mutation in *Rb1* could interfere with various interactions and post-translational modifications, among other processes [6].

It is widely accepted that the factor that triggers this type of cancer is the homozygous loss of the *Rb1* gene, according to Knudson’s two-hits hypothesis [17, 18]. However, with the tumour suppressor p53 exists strong evidence showing that a cancer phenotype can be triggered with one single mutation of p53 in one allele. Bernard et al. showed that from 3324 patients with myelodysplastic syndrome, one-third presented monoallelic mutations of p53 [19]. This is in line with other reports where heterozygous inactivation of p53 in rats develop sarcomas at eight months of age [20]. In addition, there is recent evidence in retinoblastoma tumours itself where only one allele was found mutated [21, 22].

Despite these abundant reports, none have further explored the underlying mechanisms by which RB mutants perturb cellular homeostasis. In this work, we aimed to explore the impact of a nonsense pR552* mutation and missense pN328H and pD718N mutations in the *Rb1* gene on cellular activities such as cell proliferation, migration or subcellular localization. We observed a mislocalization of all three mutants. Furthermore, the pR552* mutant showed the highest growth rate, viability, and migration compared to its wild-type counterpart, either alone or co-expressed with the wild-type. This finding raises the question of whether, similar to the tumour suppressor protein p53, it is also possible to find gain-of-function mutants in RB.

## RESULTS

### RB mutants found in retinoblastoma patients

**Figure 1:**
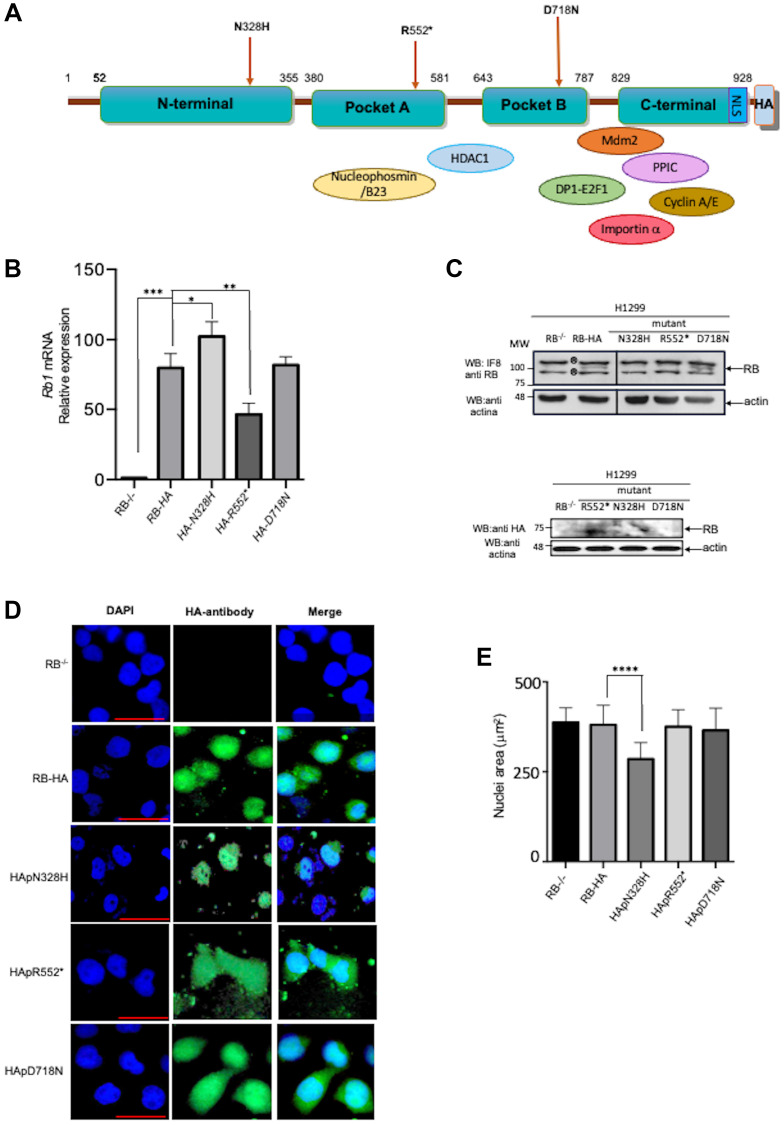
Cellular expression and localization of the three different RB mutants. (**A**) Schematic representation of the RB protein with the three studied mutations and protein interaction partners. The N-terminus contains the nuclear matrix binding domain. The combination of pocket A and pocket B forms the small pocket structure. The small pocket, along with the C-terminal domain, forms the large pocket. The pocket interacts with E2F1, Nucleophosmin/B23, HDAC1, and the transcription factor. Additionally, MDM2, Cyclin A and E, and importin α bind to the C-terminal domain. The mutants are indicated with brown arrows. (**B**) Evaluation of *Rb* mRNA expression levels in stably expressed mutant cell lines. (**C**) Western blot of RB protein expression levels in stably expressed mutant cell lines. The bands marked with ® are non-specific bands of the antibody. (**D**) Immunofluorescence analysis of RB in the mutant cell line. The localization of each mutant is depicted in green, while the nuclei are stained blue with DAPI. The scale bar corresponds to 50 mm. (**E**) The nucleus size is presented in a bar diagram. Asterisks indicate significant differences compared to the control (*p* < 0.05; Student’s *t*-test).

Retinoblastoma (RB) is a phosphoprotein consisting of 928 amino acid residues and comprising various domains. It begins with the N-terminal domain, followed by pocket A and pocket B, and concludes with the C-terminal domain (Figure 1A). The multidomain structure of this protein provides it with significant plasticity, enabling it to interact with a vast array of different partners [16, 23–26] (Figure 1A). *Rb1* undergoes mutations in several types of cancers, including retina, lung, prostate cancer, osteosarcoma, glioma, among others [6, 7, 27–30]. Here, through site-directed mutagenesis, we generated three distinct mutations identified in retinal cancer patients using an RB template labeled RB-HA, which incorporates an HA-tag at the 3′-end. One of these mutants, pR552* is also prevalent in other types of cancers (Table 1). The first mutation, pN328H, represents a conservative point mutation. According to alpha fold simulation, it exhibits a slight conformational change in the loop between pockets A and B, and a significant alteration in the C-terminal domain [31] (Supplementary Figure 1A). The second mutant is pD718N, representing a non-conservative mutation. The alpha fold simulation indicates a slight conformational change in the N-terminal loop, the loop between pockets A and B, and a pronounced change in the C-terminal domain (Supplementary Figure 1B). Lastly, we generated a mutant of pR552* that introduces a stop codon at residue R552 located in pocket A. This mutant has been identified not only in retinoblastoma but also in osteosarcoma, glioma, among other types of cancer. The alpha fold simulation suggests a slight conformational change in the N-terminal loop; it is worth noting that this mutant lacks the pocket B and the C-terminal domain (Supplementary Figure 1C). The first two mutants exhibit changes in the C-terminal region; nonetheless, they potentially interact with some of the proteins that recognize the sequence of this domain. In contrast, pR552* completely lacks this region, losing the ability to interact with a group of proteins involved in various signaling pathways, including crucial players such as E2F1 and cyclin A.

**Table 1:** RB mutant analyses with different approaches

Nucleotide position	Type of mutation	Aminoacid change	Found in
c.928A>C	Conservative aa change	N328H	Retinoblastoma
c.1654C>T	Stop codon	R552*	Retinoblastoma Osteosarcoma Glioma
c.2152G>A	Non-conservative aa change	D718N	Retinoblastoma

### Cellular expression and localization of the three different RB mutants

To investigate the phenotype of these mutations, we initially knocked out the endogenous RB protein using CRISPR/Cas in the H1299 cell line, which lacks p53 expression (Supplementary Figure 2). We chose H1299 to eliminate p53-dependent apoptosis unleashed by RB absence. First, we stably transfected the H1299 RB−/− cells with each mutant, determined the *Rb1* mRNA expression levels, and compared them with the *Rb1-HA* mRNA version and the three mutants (Figure 1B). In terms of mRNA expression, we observed that the mutant *HA-D718N* did not exhibit a change compared to *Rb1-HA*, the mutant *HA-N328H* showed increased expression levels, and finally, the mutant HA-R552* showed a significantly decreased expression (Figure 1C). In contrast, at the protein level, the mutant HApN328H show a minimal level of expression, whereas the HApD718N mutant shows a similar level of expression in comparison with the RB-HA. The HApR552* showed an important decrease in protein expression in line with the mRNA levels (Figure 1C).

Immunofluorescence assays were performed on each cell line generated for the mutants. An anti-HA-tag antibody was employed to detect each of the RB proteins. The HApN328H mutant exhibits nuclear localization similar to the wild-type RB-HA. Interestingly, this mutant shows a smaller nucleus size and cannot localize into the nucleoli. It has been shown that hyperphosphorylated RB can interact with nucleophosmin/B23 through the RB pocket A region [32]. According to the alpha fold simulation, the pN328H mutant exhibits a change in the intrinsic disorder region between pocket A and B, potentially explaining its inability to translocate to the nucleolus. Alternatively, the mutation may promote a change in the phosphorylation pattern (Figure 1A, 1D and Supplementary Figure 1A).

On the other hand, mutants HApD718N and HApR552* lose the localization pattern and are found in both the cytoplasm and the nucleus. It has been reported that the nuclear localization signal of RB is located in the C-terminal region; the mutant pD718N shows a significant change in the conformation of this region, whereas pR552* lacks the entire domain. The nucleus sizes remain identical to the wild-type RB-HA (Figure 1A, 1E and Supplementary Figure 1B, 1C).

### Cell proliferation, migration and viability

**Figure 2:**
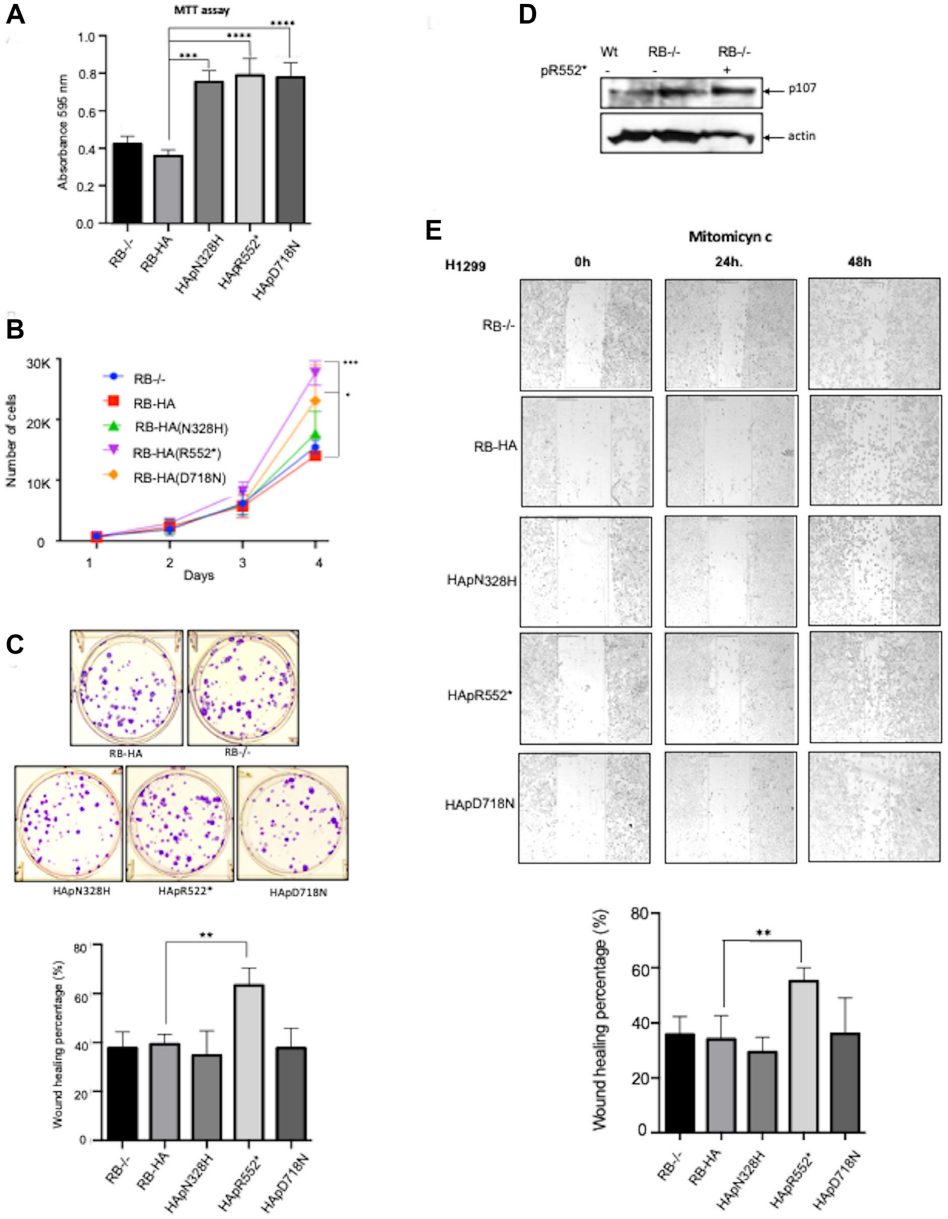
Characterization of the three mutants. (**A**) Percentage of metabolic cell activity measured with MTT in different mutant cultures represented in a bar diagram (^*^p < 0.05, ^**^p < 0.01, ^***^p < 0.001, ^****^p < 0.0001. ANOVA test). (**B**) Cell number counting during four days in different mutant cultures. (**C**) Clonogenic Assay in Six-Well Plates: Clones produced by each mutant; on the right panel the bar diagram illustrates differences in the mutants’ ability to form colonies (^*^p < 0.05. ANOVA test). (**D**) p107 is upregulated in H1299 RB−/− cells and further increased with pR552*. It is shown a western blot result where p107 is increased in H1299 RB knock out cells, the transient transfection of HApR552* seem to strengthen this upregulation. (**E**) Wound healing migration assay for each mutant: The left panel shows healing of wounds by migrated cells at 48 hours and the percentage changes in wound size are shown on the right panels. Asterisks indicate significant differences compared to the control (^*^p < 0.05, ^**^p < 0.01. ANOVA test).

To assess the proliferation rate of the stably transfected cells for each mutant, we conducted the colorimetric MTT assay. We observed that all three mutants exhibited an increase in proliferation. Notably, the mutant HApD718N and the smaller mutant HApR552* showed the highest increase in proliferation according to the MTT assay (Figure 2A). These findings were further confirmed with a cell counting assay; in this experiment, HApR552* exhibited the highest proliferation rate, more than twice that of the wild-type RB-HA (Figure 2B). HApD718N also displayed a high proliferation rate, while HApN328H showed a slight increase in proliferation, barely higher than the wild type (Figure 2B).

In addition, we performed a clonogenic assay, also known as a colony formation assay, which is based on the ability of a single cell to grow into a colony. This assay quantitatively measures the ability to undergo unlimited division and serves as an indicator of cancer cells [33, 34]. As expected, all mutant cell lines generated colonies, as H1299 is indeed a cancer cell line. Interestingly, only the mutant HApR552*, which produces a truncated protein, showed a significant increase in its ability to form colonies (Figure 2C). Notably, this specific mutant lacks the C-terminal region, thereby also losing the ability to interact with proteins as the transcription factor E2F1 and potentially the capacity to arrest the cell cycle in the G1/S phase.

Some reports [35, 36] have shown that in the absence of the wild-type RB protein, its homologous RBL1/p107 increases its levels to compensate for the loss of cell cycle control. We investigated whether, in the presence of the HApR552*, the RBL1/p107 also increased its levels. Indeed, our observations indicated that RBL1/p107 increases in the presence of HApR552* (Figure 2D). It is possible that the expression of the truncated form HApR552* could, in some way, interfere with the role of RBL1/p107 through the binding of common targets, at least via their N-terminal domains.

Finally, a wound-healing assay was employed to evaluate the migration capacity of investigated cell lines. We observed a significant increase in wound healing with the HApR552* mutant. However, none of the other mutants exhibited any effect on the migration assay (Figure 2E). Taken together, these results indicate that the HApR552* exhibits the most significant perturbation in behavior compared to the RB-HA wild-type protein. This mutant pR552* has been identified with high recurrence in different studies in Vietnamese [37], Portuguese [38], Canadian [39], American [13], English [11], and Mexican (this work) patients among others; consequently, we decided to continue studying this particular mutant.

### Effect of the HA tag on the behavior of the truncated HApR552* mutant

The truncated mutant HApR552* shows high recurrence in patients; it also exhibits the most significant impact on the cell at various levels, including localization, migration, proliferation, and viability. Consequently, we questioned whether the HA-tag plays a role in the observed effects of the mutant. To address this, we established a stable cell line with the truncated mutant lacking the HA-tag, named pR552*, and assessed its impact on the cell phenotype.

**Figure 3:**
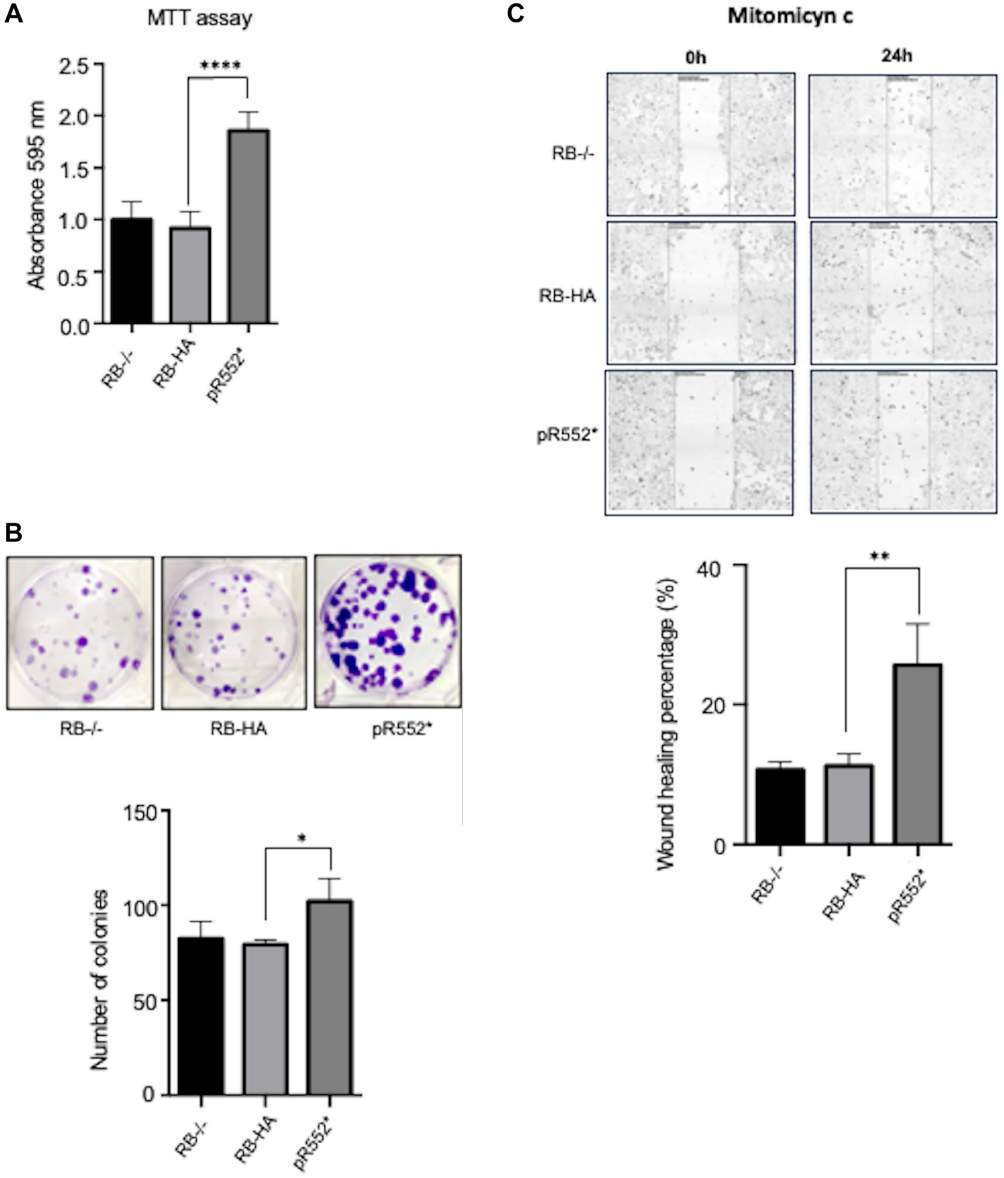
Effect of the HA-Tag on the behaviour of the truncated HApR552* mutant. (**A**) Percentage of metabolic cell activity measured with MTT in pR552* without the HA-Tag. Mutant and controls cultures are represented in the bar diagram (^*^p < 0.05, ^**^p < 0.01, ^***^p < 0.001, ^****^p < 0.0001. ANOVA test). (**B**) Clonogenic Assay in Six-Well Plates: Clones produced by pR552* without the HA-Tag. The bar diagram illustrating differences in the mutants’ ability to form colonies (^*^p < 0.05; Student’s *t*-test). (**C**) Wound healing migration assay for the pR552* without HA Tag. On the left panel is shown the migration of control cell lines compared to the cell line expressing the pR552* without the HA Tag. The percentage changes of wound healing are shown on the right panel. (^**^p < 0.01. Student’s *t*-test).

To examine proliferation, we conducted MTT experiments. Notably, the difference in proliferation between HApR552* and pR552* compared to RB-HA is highly pronounced and consistent in both cases (Figures 2A and 3A). This outcome was similarly observed in the counting-cells assay measuring proliferation. The viability of cells with the non-tagged truncated mutant was also evaluated, revealing a significant difference compared to the wild-type cell line transfected with the mutant (Figure 3B). Consistently, the pR552* showed a high percentage of wound healing rate as compared to RB-HA (Figure 3C). In conclusion, we assert that the substantial effect observed with the mutation pR552* is attributed to the mutation itself and not an artefact of the HA-Tag.

### The HApR552* mutant exhibits a gain of function behavior

**Figure 4:**
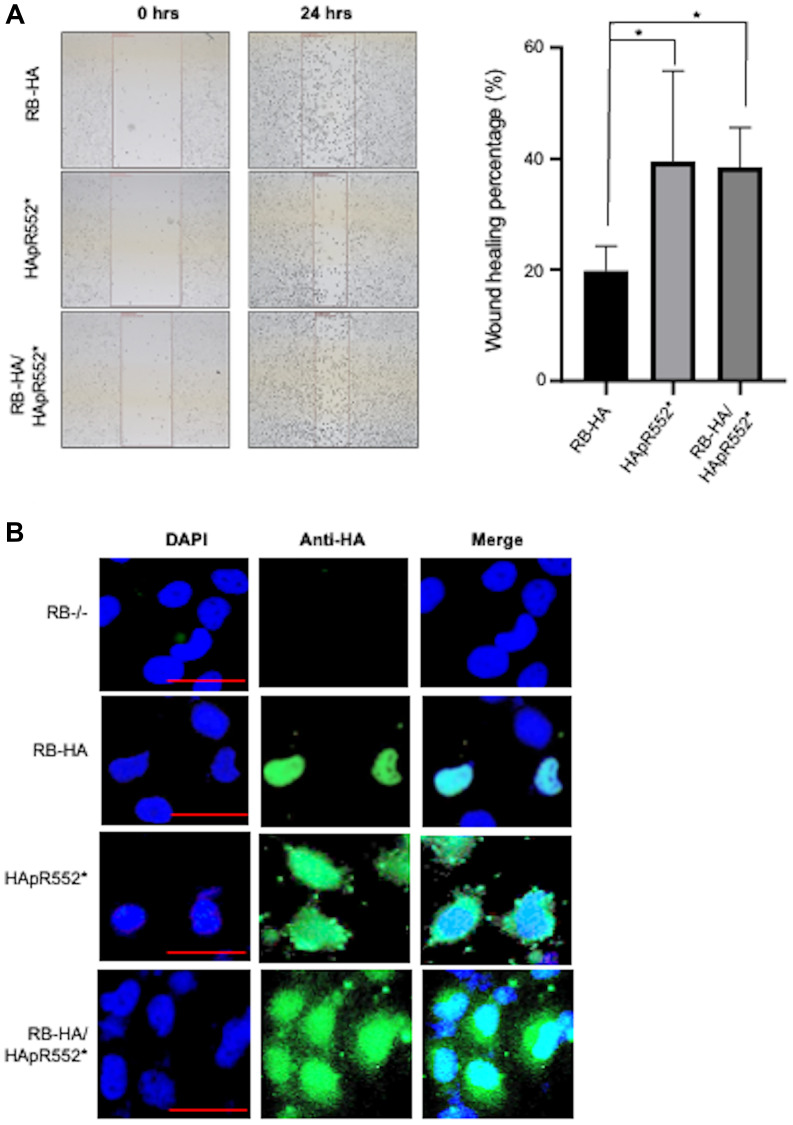
The HApR552* mutant exhibits a gain of function behaviour. (**A**) Wound healing migration assay for HApR552* mutant and wild-type RB-HA cells, as well as the co-transfection of these both constructs: The healing of wounds by migrated cells at 24 hours, and the percentage change in wound size are shown in the right panels (^*^p < 0.05; ANOVA test). (**B**) Immunofluorescence analysis of co-transfected cells with HApR552* mutant and wild-type RB-HA cells. The localization of RB-HA and the mutant is visualized using an HA antibody, depicted in green, while nuclei are stained blue with DAPI. Despite the presence of RB-HA, there is no rescue of the wild-type phenotype, as both exhibit a nucleo-cytoplasmic localization similar to the HApR552* mutant. Scale bar correspond to 50 mm.

Subsequently, we aimed to investigate whether the truncated mutant, when expressed alongside a wild-type version of the protein, maintains this phenotype or if the presence of the wild-type RB protein rescues it. To test this, we co-transfected cells with both HApR552* and RB-HA and exposed the cells to mitomycin C treatment to inhibit proliferation. Subsequently, we induced a wound in the cell culture and measured the healing process to analyse migration. Our observations revealed that the co-transfected cells demonstrated a healing percentage similar to that of HApR552* alone. Both showed higher migration than the wild-type RB protein, as depicted in Figure 4A.

Furthermore, we conducted an immunofluorescence assay using the RB-HA wild type, the HApR552* mutant, and both in a co-transfected RB-HA and HApR552* experiment. We observed the classical nuclear localization pattern in the wild-type protein, whereas the truncated mutant exhibited nuclear-cytoplasmic localization. However, in the co-transfected cells, RB maintained the localization profile seen in the mutant alone, indicating that the wild-type protein does not rescue the aberrant behavior of HApR552* (Figure 4B).

**Figure 5:**
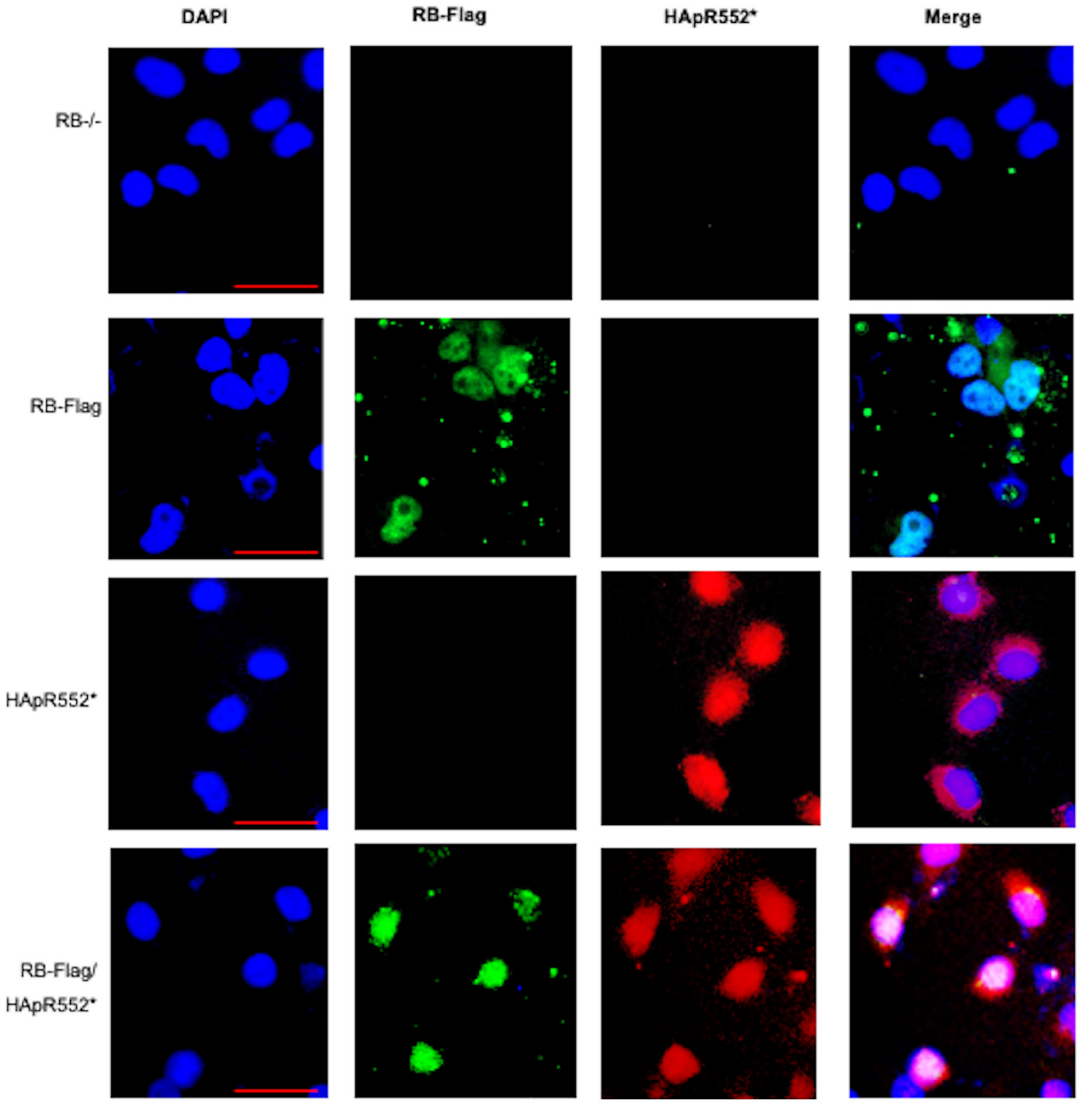
Immunofluorescence analysis of co-transfected cells with HApR552* mutant and wild-type RB-Flag cells. The HApR552* localization is visualized in red, and the RB-Flag mutant is visualized using a Flag-Tag antibody in green. Nuclei are stained blue with DAPI. RB-Flag is expressed and localized in the nucleus, while HApR552* shows a nucleus-cytoplasmic localization. Scale bar correspond to 50 mm.

Finally, we generated a construct with the wild-type tagged with a Flag-Tag, named RB-Flag to distinguish it from the wild-type HApR552*. Then, we performed an immunofluorescence assay co-transfected with both constructs and monitored the expression of RB-Flag in green and HApR552* in red. As observed in Figure 5, both constructs are expressed, and the pattern of both mutants is conserved. Surprisingly, even in the presence of the wild-type protein, the mutant allows the cells to maintain their carcinogenic characteristics, making this a gain-of-function mutant (Figures 4B, 5 and Supplementary Figure 3).

### Mexican family carried the truncated mutant pR552*

**Figure 6:**
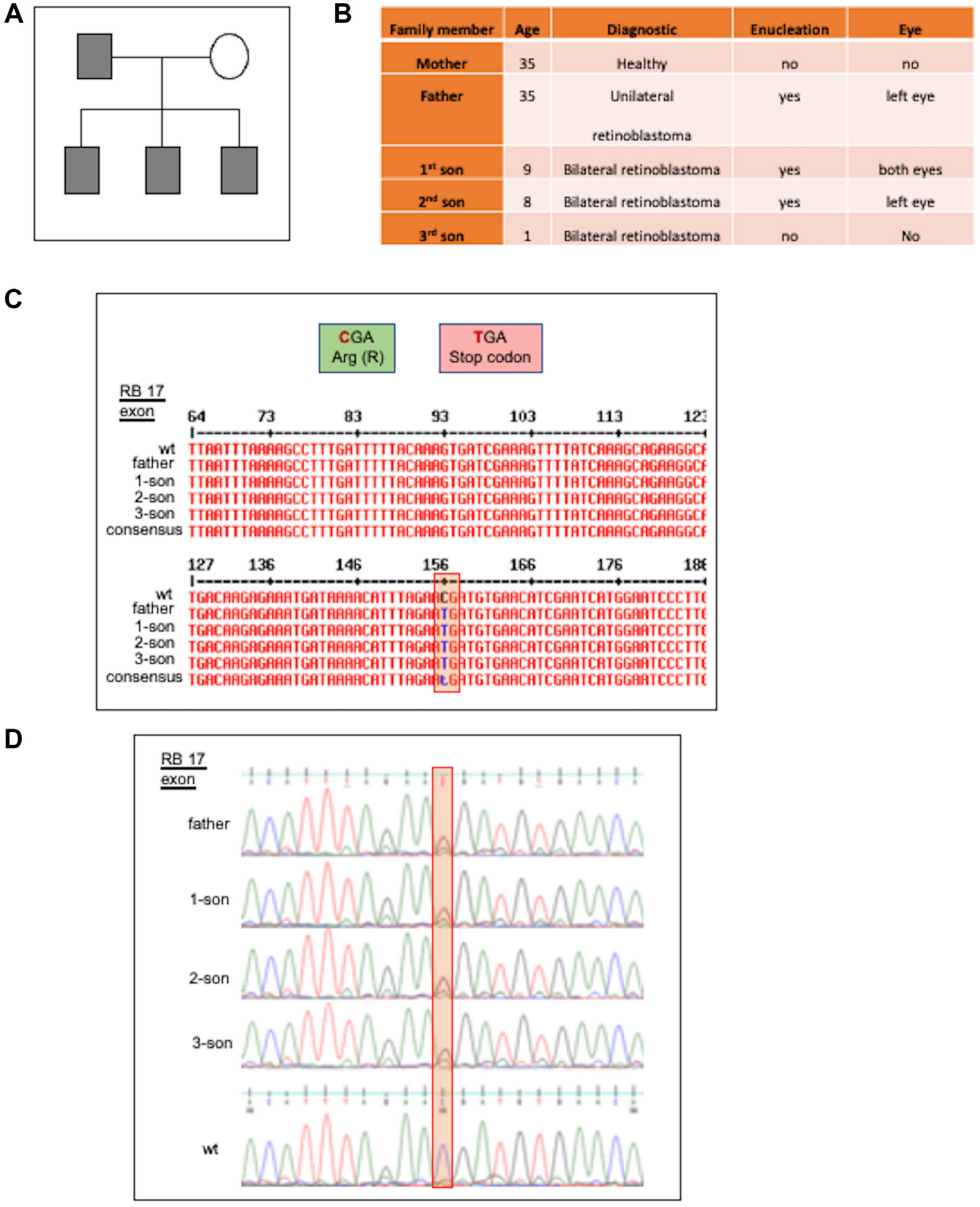
Mexican family carrying the truncated mutant pR552*. (**A**) Pedigree Analysis: The family pedigree indicates that the father and all three sons present the disease. (**B**) Data from the family members RBTR4, carried the stop codon retinoblastoma mutation pR552*. (**C**) Sequence Alignments: A portion of the 17-exon sequence of the family demonstrates the change 1654 C>T, resulting in an anticipated stop codon instead of the normal Arginine (R). (**D**) The electropherogram of the sequence of a segment of the 17-exon region of the family reveals the mutation 1654 C>T.

The above results help us to explain the behavior shown by the Mexican family carrying the pR552* mutant named RBTR4 (Figure 6A). Residing in San Luis Potosí, México; this family comprises a mother, father, and three sons aged 9, 8, and 1 year (Figure 6B). The primers used to sequence the 27 exons of RB were based on the work of Mohd-Kahlid et al. 2015 [40]. The father was diagnosed with unilateral retinoblastoma at seven months old and enucleated from the left eye. He transmitted the mutant as an autosomal dominant trait with complete penetrance and high expressivity since the three sons showed bilateral retinoblastoma. The first son underwent enucleation of both eyes at one year old, while the second one was diagnosed at the age of 22 days and enucleated from the left eye at nine months. The third son is currently under treatment and conserved both eyes. The mother remains healthy. Peripheral venous blood was used for DNA extraction, followed by sequencing of the 27 exons. This sequencing revealed the presence of the pR552* truncated mutant in all male members of the family (Figure 6C, 6D). Taken together these results strongly indicate that the truncated mutant pR552* represents a gain-of-function mutation in the retinoblastoma protein, marking the first reported instance of such a mutation.

## DISCUSSION

During the present work, we studied the phenotype of three different point mutants on the *Rb1* gene observed in retinoblastoma patients: pD718N, pN328H, and pR552* with an HA-Tag. The three mutants exhibited distinct differences in proliferation, viability, migration, and localization. While two of them were localized in both the nucleus and cytoplasm, the third, even though localized only in the nucleus, was not observed in the nucleolus. Among the three mutants, only pR552* presented notable changes in viability experiments and a significant alteration in migration, with implications for its effects. As we continued analyzing the effects of this mutant, we prepared another cell line with the truncated mutant but without the HA-Tag to avoid potential interference. The results showed that, indeed, the new cell line behaves similarly to the previous one, indicating that the presence of the HA-Tag is not a factor influencing the previously observed results.

We wonder if a wild-type protein copy could reset the wild-type phenotype. The two-hit Knudson hypothesis establishes that most tumour suppressor genes require mutations in both alleles to develop cancer [18]. A single functional tumour suppressor gene copy is enough to withstand the perturbations of the mutated counterpart. For inherited cases, the first hit comes from one of the parents and is present in the other body cells, whereas the second hit is a somatic event in the patient’s retina. Surprisingly, the mutated phenotype persisted in co-transfected assays with a wild-type *Rb1* gene, suggesting that just one hit is necessary to develop the disease.

A gain of function (GOF) mutation refers to the mutated gene product that has acquired a new molecular or biochemical function or even a new pattern of expression and is dominant or semi-dominant [41]. Many GOF mutations have been reported on different tumour suppressors, one of the most frequent being the transcription factor p53, the guardian of the genome [42, 43]. Some p53 missense mutations gain novel oncogenic activities [44–47]. However, patients carrying the mutated *Rb1* gene somehow alter the structure of the *Rb* RNA, which can result in a truncated form of the protein that has been reported to be degraded. In any case, the mutation leads to a loss of function, with no reported gain-of-function (GOF) *Rb1* mutants. The final consequence of this loss of function is continuous cell cycle progression, promoting dysregulated proliferation [37]. If this were true for the pR522* mutant, then it should not have any activity and cell proliferation parameters might be expected as the RB KO cell line. However, our results show that the pR552* truncated mutant protein is expressed; the resulting protein is truncated in the A pocket, with a size slightly more than half of the full-length RB. This portion of the protein is not only able to be expressed stably but also presents extended localization (nucleus and cytoplasm), exhibiting the highest proliferation when expressed in cells. In the migration assay, the truncated mutant pR552* also displays the highest activity compared with the wild-type RB protein and the other two mutations, pN328H and pD718N. We conclude that the pR552* mutant behaves as a GOF with a dominant phenotype (Figures 4 and 5).

These results contribute to explaining the behavior of retinal cancer in the RBTR4 family, where the truncated mutant exhibits complete penetrance and high expressivity. While the father showed a unilateral presentation, all three sons displayed bilateral diseases. Another surprising observation is that typically, if either the mother or father has a mutated gene, the child has a 50% chance of inheriting that mutated gene. However, in this family case, the father transmitted the mutant pR552* to 100% of his children.

## MATERIALS AND METHODS

### Patients

Patients involved in the study were part of the approved protocol, overseen by the research and ethics committee at the Central Hospital “Ignacio Morones Prieto”, San Luis Potosí, SLP, Mexico. Diagnostic and therapeutic manoeuvres were conducted following the Official Mexican Standard NOM-012-SSA3-2012, which outlines the criteria for executing research projects related to human health.

Inclusion criteria: Children with diagnosis of retinoblastoma, without a known diagnosis of other cancer. Exclusion criteria: Patients with diagnosis of other type of cancer. Controls: Healthy children at pediatric age and healthy adults in the same range of age of the family member patients.

### Cell lines and mutants

The experiments were performed using the human H1299 cells (non-small lung carcinoma cell line) and H1299 RB−/− cells. The cells were grown in RPMI 1640 medium supplemented with 10% fetal bovine serum, L-glutamine 2 mM, penicillin 100 U/ml and streptomycin 100 μg/ml (Invitrogen). H1299 RB−/− was stably transfected by antibiotic selection with RB-HA wild-type, RBN328H, RBR552* and RBD718N. The full-length cDNA for human *Rb* was subcloned into the *Hin*dIII and *Bam*HI sites of the pcDNA3.1 (RRID:Addgene_14743) vector with the previous addition of the label hemagglutinin (HA) tag in the C-terminal of RB. All mutants were generated by site-directed PCR mutagenesis using the previous Rb-HA template. Then RB-HA wild-type or mutants were released and subcloned in the pLNCX2 (RRID:Addgene_89818) retroviral vector with *Hin*dIII and *Not*I sites. Purified DNAs were used to produce retroviruses in HEK293T (RRID:CVCL_0063) cells by transiently transfecting the RB-HA wild-type or mutants together with Gag-pol (encodes for capsid proteins, reverse transcriptase and integrase) and VSV-G (encodes for the vesicular stomatitis virus G envelope protein) expressing plasmids, cells were transfected by lipofectamine 2000 reagent. Supernatants from transfected cells containing retroviral particles were collected 48 h after transfection and used to infect H1299-RB^−/−^ cells in the presence of 8 μg/ml of polybrene for 8 h. 24h after infection cells were selected by adding G418 to the growth medium for at least 5 days. In addition, the full-length cDNA for human RB was subcloned into the *HindIII* and *BamHI* sites of the c-Flag pcDNA3.1 vector.

### CRISPR/Cas9

The H1299 RB−/− cell line was built using the CRISPR Cas9 system. H1299 (RRID:CVCL_0060) cells were cotransfected with the RB CRISPR/Cas9 KO plasmid (Cat. No. sc-400116, Santa Cruz Biotechnology, Santa Cruz, CA, USA) and the RB HDR plasmid (Cat. No. sc-400116-HDR Santa Cruz Biotechnology, Santa Cruz, CA, USA). The RB CRISPR/Cas9 KO plasmid encodes for a guide RNA and nuclease, and the RB HDR plasmid encodes a homology-directed DNA repair template corresponding to the cutting sites generated by the RB CRISPR/Cas9 KO plasmid and a puromycin resistance gene to enable the selection of stable knockout cells. The single-celled clones were obtained by serial dilution and cultured with 3 μg/ml of puromycin (Cat. No. P7255, Sigma-Aldrich). The clones were tested for RB expression through Western blot, and only the negatives for RB protein expression were amplified.

### Western blot analysis

Each cell line was lysed using RIPA lysis buffer (200 mM NaCl, 0,2% NP-40, 10% (v/v) glycerol, 1 mM dithiothreitol (DTT), 1 mM EDTA and 25 mM Tris-HCl, pH 7,8) with a 1% protease inhibitor cocktail (Cat. No. 4693132001, Sigma-Aldrich). Equal amounts of proteins were loaded into SDS-PAGE (polyacrylamide gel electrophoresis with sodium dodecyl sulfate) at 8%. The protein gels were transferred to a BioTrace NT nitrocellulose membrane (PALL Corporation) and blocked with 5% fat-free milk at PBS pH 7,4. The proteins were probed with the corresponding antibodies: anti-RB IF8 mouse (Cat. No. sc-102 Santa Cruz Biotechnology, CA, USA), anti-HA mouse (Cat. No. ab130275, Abcam, USA), and anti-actin-HRP (Cat. No. AC-15, Sigma-Aldrich).

### RT-qPCR

The mRNA for each cell line was extracted using the TRIzol reagent. Reverse transcription to cDNA was performed using M-MuLV (Cat. No. N01-M0253S, New England Biolabs) reverse transcriptase with 1 μg of RNA. PCR amplification was performed in triplicate using the following primers: for human GAPDH, 5′-TCC AAA ATC AAG TGG GGC GA-3′ and 5′-TGA TGA CCC TTT TGG CTC CC-3′; for RB 5′CCT CTC GTC AGG CTT GAG TTT-3′ and 5′-GCT CTC TCT CTG ACA TGA TCT GG-3′. Each PCR mixture contained 1 μl of cDNA, 1ul of each primer (10 μM) and 5 μl of the SYBR Green (Thermofisher-K0221) in a final volume of 10 ul. The RT-qPCR was performed on 96-well microtiter plates using the 7500 Fast (Applied Biosystems) equipment. The amount of *Rb* mRNA (Ct of Rb) was normalized by subtracting the amount of GAPDH mRNA (GAPDH ct). The relative amount of *Rb* mRNA was obtained using the value 2^−ΔΔCt^.

### Immunofluorescence

3 × 10^4^ cells/well were seeded in a 24-well plate with a round coverslip for each cell line and incubated for 24 hours. Briefly, the cells were fixed with 3,7% paraformaldehyde for 30 min and permeabilized with PBS/Tritón X-100 (0.5%) for 1 h. Afterwards, the cells were washed with phosphate-buffered saline (PBS) for 5 min, followed by a 30 min incubation with PBS with bovine serum albumin (BSA) 3% to block. After blocking, the cells are incubated overnight at 4°C with 50 ul of the primary anti-HA mouse (Cat. No. ab130275, Abcam, USA) antibody at a dilution 1:100 prepared in PBS/BSA. Three washes of 5 min are done with 1X PBS and then incubated for 1 hour at 4°C with the secondary anti-mouse antibody conjugated with Alexa Fluor 568 (Cat. No. F0257, Sigma-Aldrich) at a dilution of 1:1000 prepared in PBS/BSA solution. Afterwards, three washes of 5 min are made with 1X PBS at room temperature. Subsequently, the coverslips are assembled with 4 ul of the mounting solution containing 4′,6-diamidino-2-phenylindole (DAPI). The cells were photographed under a fluorescence microscope.

An additional experiment was conducted where 5 × 10^4^ H1299 RB−/− cells were seeded per well in a 24-well plate with round coverslips and incubated for 24 hours. After incubation, they were co-transfected with RB-Flag and RB(R552*)-HA and incubated for 48 hours. Cells were fixed with 3.7% paraformaldehyde for 30 min and permeabilized with PBS/Tritón X-100 (0.5%) for 1 h. Afterwards, the cells were washed with PBS for 5 min, followed by a 30-minute incubation with PBS with 3% BSA to block. After blocking, cells are incubated overnight at 4°C with a mixture of the primary antibodies, anti-HA rabbit (Cat. No. ab236632, Abcam) at dilution of 1:100 and anti-Flag mouse (Cat. No. F1804, Sigma-Aldrich) at dilution of 1:100 prepared in PBS/BSA. Three 5 min washes are performed with 1X PBS and then incubated for 1 hour at room temperature with a mixture of the secondary antibodies: anti-mouse antibody conjugated with Alexa Fluor 568 (Cat. No. F0257, Sigma-Aldrich) 1:100 and anti-rabbit antibody conjugated with Rodamina (Cat. No. ab6718, Abcam, USA) 1:1000, prepared in PBS/BSA solution. Afterwards, three 5 min washes are performed with 1X PBS at room temperature. Subsequently, the coverslips are assembled with 4 μl of the mounting solution containing 4′,6-diamidino-2-phenylindole (DAPI). The cells were photographed under a fluorescence microscope.

### MTT assay

1 × 10^4^ cells/well were seeded in a 96-well plate in 100 ul of supplemented RPMI culture medium for each cell line and incubated for 48 hours. The MTT assay (3-(4,5-dimethylthiazol-2-yl)-2,5-diphenyltetrazolium bromide) was performed using the MTT Assay Kit (Cell Proliferation) (ab211091) according to the manufacturer’s protocol. Briefly, the MTT protocol used consisted on replacing the medium in which the cells are with a serum-free medium and MTT reagent (tetrazolium dye solution) and incubating them for 3 hours at 37ºC. After incubation, the cells were treated with MTT solvent for 15 minutes at room temperature. Absorbance was measured at OD = 595 nm.

### Colony formation assay

Cells were cultured in a 6-well plate with a density of 200 cells/well density in 2 ml of supplemented RPMI culture medium and incubated for 10 days. The cells were then fixed with 100% methanol for 10 minutes of incubation at room temperature, and then the colonies formed were stained with 0.5% violet crystal solution for 10 minutes at room temperature. To remove the background stain, the cells were washed twice with ddH2O. Finally, the plaque was left to dry, and the colonies were photographed and counted with the “ColonyCount” application.

### Wound-healing assay

7 × 10^4^ cells/well were seeded in a 24-well plate in 500 ul of supplemented RPMI cultured medium and incubated for 24 hours. Cells were washed with 1X PBS and treated with mitomycin c (10 μg/ml for 1 hour) in a serum-free medium. After incubation, cells were again washed twice and left with 1% serum-supplemented medium. The cells were photographed at 0, 24 and 48 h to compare the migration capacity of the cell lines. Zen Lite 2012 microscopy and fluorescence software were used to analyse and quantify closure rates.

### Statistics

Statistical analyses were performed using the GraphPad Prism 8.0 program (RRID:SCR_002798). Unidirectional analysis of variance (ANOVA) followed by the Bonferroni test was used to analyse the statistical differences between the groups. The results of the statistics were presented as a mean ± standard error of the mean (SEM). The *p*-value less than 0.05 was accepted as statistically significant (^*^p < 0.05, ^**^p < 0.01, ^***^p < 0.001).

## SUPPLEMENTARY MATERIALS



## Abbreviations

*Rb1*: Retinoblastoma gene
RB: Retinoblastoma protein
HApD718N: mutated protein D718N with a HA tag
HApR552*: mutated protein R552* with a HA tag
HApN328H: mutated protein N328H with a HA tag
pR552*, pN328H and pD718N: mutate proteins without a tag
