# Supplementary material for: Analysis of pathogenic variants in retinoblastoma reveals a potential gain of function mutation
Source: Genes Cancer. 2025 Jan 20;16:1–15. doi: 10.18632/genesandcancer.239 (PMC11748778; doi:10.18632/genesandcancer.239)
Supplement: Supplementary file 1 [file ganc-16-239-s001.pdf]

Analysis of pathogenic variants in retinoblastoma reveals a potential gain of function mutation

SUPPLEMENTARY MATERIALS

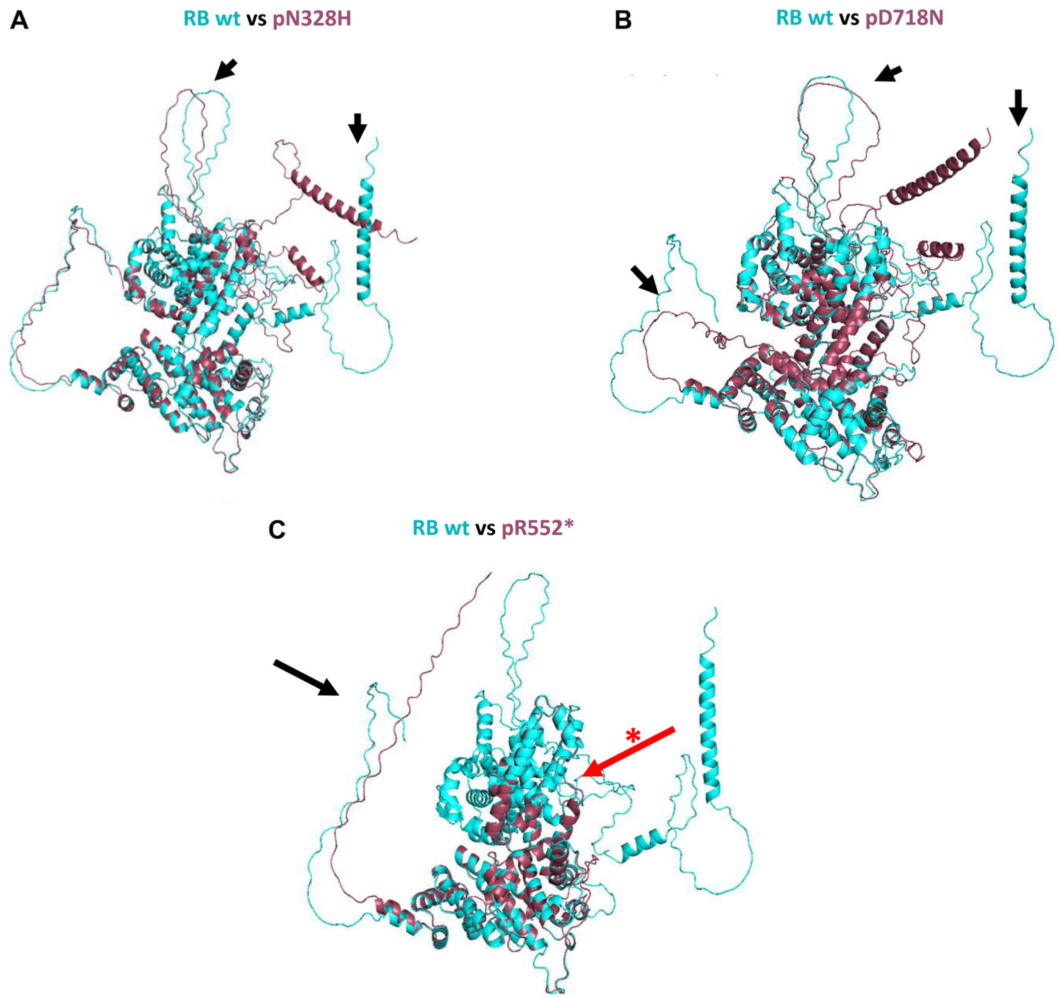

Supplementary Figure 1: (**A**) AlphaFold molecular modelling alignment of the RB wild-type (cyan) and mutant pN328H (red). The arrows indicate that the main differences lie in the C-terminal domain and between pockets A and B. (**B**) AlphaFold molecular modeling alignment of the RB wild type (cyan) and mutant pD718N (red). The arrows indicate the main differences in the C-terminal domain, the N-terminal intrinsically disordered region, and between pockets A and B. (**C**) AlphaFold molecular modeling alignment of the RB wild type (cyan) and mutant pR552* (red). The black arrow indicates a difference in the N-terminal intrinsically disordered domain, and the red arrow indicates where the mutation ends. The models are presented using PyMOL (DeLano W.F. 2002 and DeLano W.F. 2004).

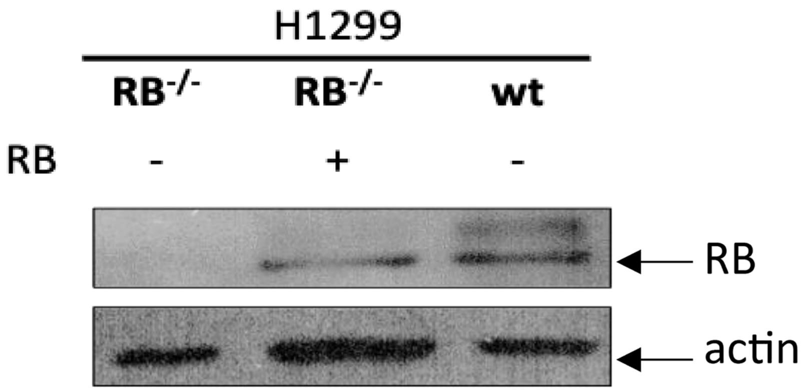

Supplementary Figure 2: Western blot of RB protein expression levels H1299 cell line wild-type and RB knock out (RB−/−), transfected or not with RB.

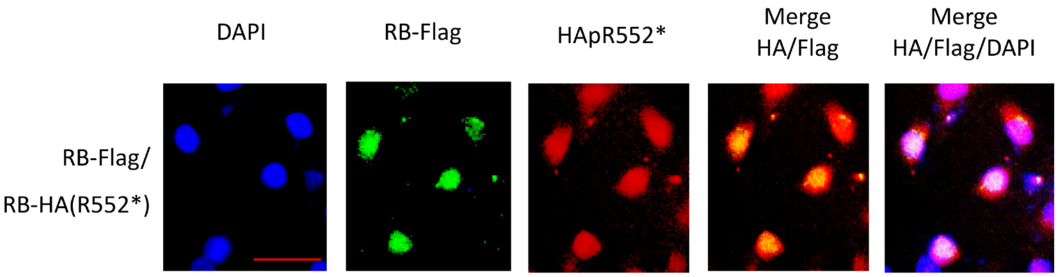

Supplementary Figure 3: Immunofluorescence analysis of co-transfected cells with HApR552* mutant and wild-type RB-Flag cells. The localisation of HApR552* is visualized in red, and the RB-Flag mutant is visualized using a Flag-Tag antibody in green. Nuclei are stained blue with DAPI. RB-Flag is expressed and localised in the nucleus, while HApR552* shows a nucleus-cytoplasmic localisation. Scale bar correspond to 50 mm.
